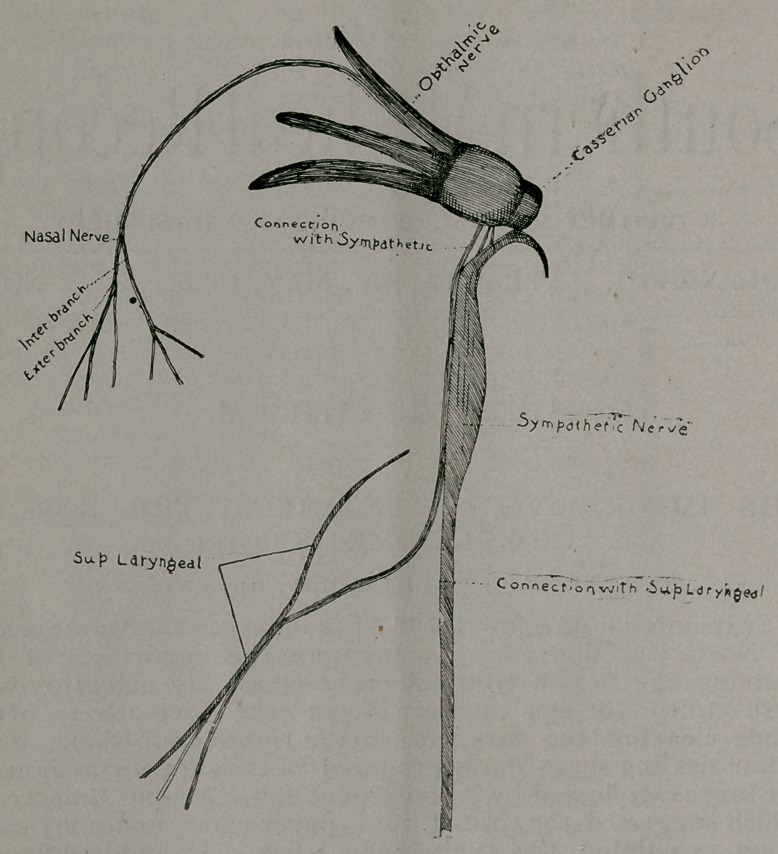# The Importance of Examining the Nose in Troublesome Coughs

**Published:** 1898-05

**Authors:** Alfred C. Palmer

**Affiliations:** Richmond, Va.


### Southern Medical Record.

A MONTHLY JOURNAL OF MEDICINE AND SURGERY.

VOL. XXVIII. ATLANTA, GA., MAY, 1898. NO. 5.

### Original Articles.

### THE IMPORTANCE OF EXAMINING THE NOSE IN TROUBLESOME COUGHS.

By ALFRED C. PALMER, M.D , Richmond, Va.

At its annual meeting in 1887 I read before the State Society of North Carolina a brief paper upon the importance of examining the *ears* in troublesome coughs. My attention was first called to this subject from the fact that often while cleaning the ears with cotton-tipped probes, etc., a peculiar tickling sensation is produced in the superior region of the larynx, followed by a desire to cough. At that time a case which suggested the title of that paper came under my care. Upon examining the tympanum, I found it highly inflamed and so sensitive to the touch that it could not be approached in the most delicate manner without throwing the patient into an attack of violent coughing. Treatment was applied entirely to the ear trouble and the cough at one disappeared. Since that time many cases have been added to the affirmative side of the list.

Having become fully convinced that there must be some nerve connection between the ear and the larynx, I undertook in that article to trace the same; first through Jacobson's nerve and its connection with the glosso-pharyngeal at its petrous ganglion, then through the fibers of that ganglion which unite with the pneumogastric, then through the pneumogastric downward to its inferior ganglion, from the middle of which is given off the superior laryngeal which supplies the sensitive mucous membrane of the superior portion of the larynx.

One investigation leads to another, and I have for some time been convinced that through similar nervous connection many distressing coughs are directly due to disturbances in the anterior nasal cavity as well as in the aural, and that they are entirely independent of any local laryngeal, bronchial or pulmonary causes and are of a purely reflex nature. The sudden expulsion of air from the lungs in the act of sneezing should suggest to our minds that an irritation in the nose has a direct influence over the expiratory nerves. In this case the irritation is primarily in the nasal mucosa, and is manifested at the other end of the line. We frequently notice, as in the examination of the ears, that the simple cotton probang which we apply to this nasal region, for the purpose of removing accumulation, making medicinal applications, etc., causes our patient to begin at once to manifest laryngeal disturbances to a greater or less degree. Very frequently we see no evidences of it at all. This is due, I believe, to the fact that the power to resist reflex influences is very much greater in some individuals than it is in others.

In tracing what I take to be this nerve connection between the nose and the larynx, I will not consume time in dilating upon their deep cranial origins, but beg leave to take as a starting point the Cæsarian ganglion. The ophthalmic branch of the fifth rises from the upper portion of this ganglion. It is a short flattened band about an inch in length, which passes forward along the outer wall of the cavernous sinus, below the other nerves, and just before entering the orbit, through the sphenoidal fissue, divides into three branches, frontal, lachrymal, and nasal. It is the last mentioned branch that directly concerns our investigation. This nerve is intermediate in size and is between the frontal and lachrymal, and is more deeply located. It enters the orbit between the two heads of the external rectus, passes obliquely inward across the optic nerve beneath the levator palpebraum and superior rectus muscles to the inner wall of the cavity where it enters the anterior ethmoidal foramen immediately below the superior oblique; it there enters the cranial cavity, traverses a shallow groove on the front of the cribriform plate of the ethmoid bone and passes down through the slit beside the crista galli into the nose, where it divides into two branches, an internal and an external. The internal branch supplies the mucous membrane of the septum near its front, while the external descends in a groove on the inner surface of the nasal bone, sending its filaments to the mucous membrane covering the fore part of the outer wall of the nostril as far as the inferior spongy bone. It then passes out of the nasal cavity to the external integuments in the region of the ala and tip of the nose. So much for the nasal nerve and its connection with the semilunar ganglion.

Starting again at this ganglion, we find on the inner side filaments connecting it with the carotid plexus of the sympathetic; passing down the sympathetic beyond its superior, outer, inner, inferior, and external branches, we arrive at its internal, which are the pharyngeal, laryngeal and the superior cardiac nerves. The pharyngeal with branches of the pneumogastric, glosso-pharyngeal and the external laryngeal form the laryngeal plexus, while the laryngeal unites with the superior laryngeal, which, as I stated before, supplies the sensitive mucous membrane of the superior laryngeal cavity.

Bearing in mind these connections, which I have passed over so hurriedly, we readily see that the mucous membrane covering the anterior region of the nasal cavity is directly in touch, so to speak, with the larynx—first through the nasal nerve, then the ophthalmic, then through the Cæsarian ganglion to the sympathetic, down this nerve to the laryngeal, on to the superior laryngeal and its terminal filaments. After satisfying ourselves on this point and observing the physiological effects which an irritation sometimes produces in the nasal cavity we must conclude that the many disorders to which this region is subjected may be attended by laryngeal disturbances. These coughs, as a rule, are of a distinctly harsh, irritating nature with scanty secretion.

I have in my care at present two cases which present typical examples of just this class of troubles. One, a gentleman, recommended to my care by Dr. Winfree, of Richmond, Virginia, of robust physique who has been troubled more or less for years with a most distressingly harsh, dry cough, purely laryngeal. There is entire absence of any pulmonary or bronchial complications. He has received treatment at many different times in various quarters. His uvula has been amputated, nasal bodies have been frequently cauterized with galvano-cautery, applications have been made repeatedly to post-nasal space. Upon examination we found an exceedingly hyperesthetic condition of the anterior nares on both sides; on the right we found a scar just inside of the nostril on its external aspect which extends from some distance upwards down to the junction of the skin and mucous membrane. He tells me that he thinks it is a result of a cauterization and it has very much that appearance. Owing to its rough surface it afforded excellent lodgment for the accumulation of mucous, which became dry, forming an irritating scab or crust. In the other nostril we discovered on the septum a small area of unhealthy tissue with crust formation. I am not thoroughly convinced but that in the process of healing after the cauterization on the right side some of the filaments of the nasal nerve became confined in the scar. With constant care and the application of various remedies the surfaces have become smooth and the accumulations less and *a priori* the cough has much improved.

The other case is a lady, sent me by Dr. Michaux, of Richmond, Virginia, whose general health has become much impaired, no doubt, from loss of proper rest at night on account of a continuous irritating cough. We found in this case in the region of the distribution of the internal branch of the nasal nerve upon both sides of the septum an area of denuded mucous membrane containing these crusts of inspissated mucous. After healing these spots and giving her remedies to keep the parts clean and moist, the cough is gradually disappearing.

These proofs are so conclusive to my mind, that I make a careful examination of every case that comes under my observation both of tympanum and the nasal cavity. I produce irritation in both by the use of the cotton-tipped probe and pay attention to the effect it produces upon the larynx.

It is very probable that you will find in all of these cases congestion of the superior laryngeal cavity, but I regard this as a result more than as a cause, and due to the fact that the parts do not find time to resume their normal condition between the attacks of coughing. Hoarseness is also present in some instances due to this congestion. The application of instruments, as well as the remedies used in the treatment, has a tendency to good, by making the mucous membrane more tolerant to the presence of irritating bodies. In the first case mentioned, in connection with the opinion that some filaments of the nasal nerve may be confined in the scar located in the right nostril, it impresses me that we should be cautious in the use of the galvano-cautery when treating nasal troubles. Scars cause disturbances in other parts of the body, and I do not see why the nasal cavity should be exempt.

I do not claim originality in presenting these conclusions, but the limited amount of literature I have been able to procure upon the subject deals entirely with posterior parts of the turbinate bodies and naso-pharynx. Viewing the matter from an anatomical standpoint, I can not see how these regions can be as fruitful a source of "reflex coughs" as the anterior surfaces mentioned above.

## Figures and Tables

**Figure f1:**